# Linking Microbial Community Succession With Substance Transformation in a Thermophilic Ectopic Fermentation System

**DOI:** 10.3389/fmicb.2022.886161

**Published:** 2022-05-04

**Authors:** Ping Wen, Yueqiang Wang, Wenfeng Huang, Weiwu Wang, Tao Chen, Zhen Yu

**Affiliations:** ^1^National-Regional Joint Engineering Research Center for Soil Pollution Control and Remediation in South China, Guangdong Key Laboratory of Integrated Agro-Environmental Pollution Control and Management, Institute of Eco-Environmental and Soil Sciences, Guangdong Academy of Sciences, Guangzhou, China; ^2^Environmental Research Institute, School of Environment, Guangdong Provincial Key Laboratory of Chemical Pollution and Environmental Safety and MOE Key Laboratory of Theoretical Chemistry of Environment, South China Normal University, Guangzhou, China

**Keywords:** continuous thermophilic environment, ectopic fermentation system, Py-GC/MS, substance transformation, thermotolerant bacteria

## Abstract

Ectopic fermentation system (EFS) is an effective technology for treating mass livestock manure. However, the associations between microbial communities and substance transformation remain controversial. This study aimed to investigate chicken manure EFS lasting 170 days using 16S rRNA sequencing and electrochemical, spectroscopic, and chromatographic analyses. The results showed a noticeable transformation of protein-like substances into humus-like substances. Meanwhile, the electron–accepting capacity increased persistently, effectively reflecting the humification of organic substances. The contents of phenols that promoted electron transfer continued to increase from 2.80 to 6.00%, which could be used as a maturity indicator for EFS. During the heating period, the dominant microbial communities were *Chloroflexi* and *Proteobacteria*, whereas thermotolerant bacteria *Cyanobacteria* and *Planctomycetes* were significantly enriched from 1.64 to 50.15% during the continuous thermophilic period of EFS. The correlation analysis manifested that these thermotolerant bacteria were the major functional bacteria for the formation of phenols and the key to driving the humification of organic substances. This study provides insights into understanding the humification mechanisms and implementing regulatory strategies in EFS.

## Introduction

With the development of the livestock industry, the excessive discharge of chicken manure poses a serious risk to the ecological system, such as nitrogenous malodor gas emission and pathogen reproduction (Young et al., 2016; Chen et al., 2019, 2020). Composting, incineration and anaerobic digestion were the common approaches for treating chicken manure (Rawoteea et al., 2017), while the application was limited by secondary pollution (Guo et al., 2015; Yang et al., 2018). How to deal with the chicken manure effectively and in an eco-friendly manner has become an urgent problem to be solved.

Ectopic fermentation system (EFS) is a dynamic system composed of bedding litters, manure and thermophilic inoculants (Yang et al., 2018). As an *ex situ* decomposition technology, EFS has gained increasing concern from applied researchers and engineers with the characteristics of large-scale disposal of organic solid wastes, especially in the scope of microbial inoculant and nitrogen transformation (Guo et al., 2013; Borhan et al., 2014; Chen et al., 2021). Guo et al. (2013) demonstrated that EFS inoculated with thermophilic strains contributed to higher temperatures and disposed more wastes. The changes in functional bacteria related to nitrogen metabolism were observed in EFS, revealing the effect of functional bacteria on nitrogen conservation (Yang et al., 2019). Chen et al. (2021) indicated that EFS had diverse microbes and clusters of orthologous groups for ammonia removal.

During the fermentation process, mineralization, and humification are the two major pathways of substance transformation (Qi et al., 2019). In this sense, the degradation and polymerization of substances are the main themes during the fermentation process, which may be accompanied by changes in the molecular weight, composition characteristics, and structural properties of substances. He et al. (2019) found that the formation of quinones in medium–molecular weight (MMW) and tyrosine in lower–molecular weight (LMW) during composting greatly contributed to the electron transfer capacity of organic matter, thereby promoting maturity. Liu et al. (2021) stated that the hyperthermophilic environment accelerated the transformation from protein-derived structures to humic acids. In addition, as a powerful driver of substance transformation, microorganisms participated in the degradation of organic matters such as reducing sugars, phenols, and protein derivatives, thus playing a vital role in the transformation of substances (Wu et al., 2017; Yu et al., 2018; Zhong et al., 2018; Yang et al., 2019). The hyperthermophilic environment promoted the formation of quinone-like substances and the degradation of tryptophan-like substances (Huang et al., 2021). Zhu et al. (2021) also reported that thermophilic bacteria were potentially involved in the biodegradation of lignocellulose. In EFS, previous studies focused on the evolution of microbial communities and the mechanisms of the nitrogen cycle (Chen Q. Q. et al., 2017; Chen et al., 2021). However, the associations between substance transformation and microbial communities remain controversial, and the associations on maturity during EFS is still unknown.

This study aimed to: (1) explore the change in substance transformation during EFS detected using electrochemical workstation, gel permeation chromatogram, excitation–emission matrix–parallel factor (EEM–PARAFAC) and PY-GC MS analyses; (2) investigate the succession in microbial communities during EFS using 16S rRNA sequencing; (3) and identify the correlation between substance transformation and microbial communities in EFS. This study could provide the theoretical and practical foundation for regulating the transformation of substances in EFS.

## Materials and Methods

### Ectopic Fermentation System Setup and Sample Collection

The chicken manure treatment plant was located in Yangjiang, Guangdong, China. The fermentation compartment dimension was 75 m in length, 4 m in width, and 1.65 m in height. The padding was composed of rice husk and 1% thermophilic inoculants with about 1.50 m in height, and thermophilic inoculants were not added subsequently. The thermophilic inoculants were derived from Guangzhou Baijia Biotechnology Co., Ltd., Guangdong, China. Further, 5 t of fresh chicken manure was spread on the padding daily and mixed thoroughly using a tipping machine. The samples were collected on days 0, 32, 64, 100, and 170 at a 30–70 cm depth following the muti-point sampling method. The mixed samples were divided into three parts. Some samples were stored at 4°C; others samples were dried and ground to 60 mesh for the physico–chemical analysis; and the remaining samples were stored at –20°C for DNA extraction.

### Physico–Chemical Parameter Analyses During Ectopic Fermentation System

The temperature was monitored regularly at five fixed points using the WSS301 project pointer thermometer (Zhongyi meter, Changzhou, China). The basic physico–chemical parameters and maturity indices such as pH, moisture, OM, dissolved organic carbon (DOC), total organic carbon (TOC), total nitrogen, C/N, and germination index (GI) were measured following the protocol proposed by Yu et al. (2018) and Huang et al. (2021). The GI was measured described previously (Meng et al., 2017). Briefly, 2 g of the sample was dissolved in 20 mL Milli–Q water and then shaken in a horizontal shaker at 25°C for 1 h. Subsequently, the extract and seeds were added to the plates and incubated in a biochemical incubator at 25°C for 3 days. The Milli-Q water cultivation was used as blank control. Electron accepting capacity (EAC) and electron donating capacity (EDC) were measured using electrochemistry workstation CHI1660 (Chenhua Co., Ltd., Shanghai, China), and a detailed protocol was referred to Yuan et al. (2012).

### Gel Permeation Chromatography

The extract solution was filtered through a 0.45 μm filter membrane before injection. The gel chromatograph analysis was executed on an Agilent 1260 infinity liquid chromatograph incorporated with a laser light–scattering detector at 254 nm (Li et al., 2014). The Milli–Q water was used as the mobile phase, and eluted at a flow rate of 1 mL min^–1^. The overlapping peaks were analyzed by peak–fitting technique. Due to the limitation of pores, the evolution of large molecule substances was faster than small molecule substances. Higher–molecular weight (HMW), MMW and LMW fractions were classified according to retention time less than 6 min, 6–8 min and more than 8 min, respectively, following the procedure described by He et al. (2019).

### Parallel Factor Analysis

As reported by Chen Y. K. et al. (2017), dissolved organic matter (DOM) was extracted by sample and Milli--Q water at a 1:10 (*w/v*) ratio for 24 h in a horizontal shaker. The DOC of the DOM was measured using a TOC analyzer (Shimadzu Corporation TOC--L, Japan). The excitation--emission matrix (EEM) fluorescence spectra were executed on the DOM using a F-7000 fluorescence spectrophotometer (Hitachi, Japan) and recorded in the excitation and emission wavelength range of 200--500 and 250--550 nm, respectively. The MATLAB 7.0 (Mathworks, United States) and DOMFluor toolbox^1^ were applied for PARAFAC analysis. Based on the PARAFAC models, two–seven components could be calculated from the EEM fluorescence spectra. The maximum fluorescence intensity (*F*_max_; a.u.) value reflects the representative concentration scores of each identified component (Ishii and Boyer, 2012).

### PY-GC MS Analysis

PY-GC MS analysis was conducted using a PY2020D–type Double–Shot Pyrolyzer coupled with an Agilent 7890–5977A GC MS system (United States). The method parameters were adapted from the study by Schellekens et al. (2017). In brief, 0.50 mg of dried sample was weighed in a crucible capsule and heated at 500°C for 1 min. The injection temperature was setting at 280°C and the heating procedure was as follows. The initial temperature was 50°C, maintained for 1 min. Subsequently, it was increased to 300°C at a rate of 7°C min^–1^ and then held for 10 min. The pyrolysis products were separated on a DB–5 MS column (Restek, 30 m × 250 μm; film thickness, 0.25 μm) with a split ratio of 1:20. The MS (70 ev) was scanned in the range of 50–600 m/z.

### Bacterial Community Analysis

According to the protocol, genomic DNA was extracted from 250 mg sample using MN NucleoSpin 96 Soi Kit (United States). The DNA concentration and quality were checked using a NanoDrop ND-2000 (Thermo Fisher Scientific, NC, United States) spectrophotometer and gel electrophoresis, respectively. The 16S rRNA amplification was performed using a specific primer: 338F (5′-ACTCCTACGGG AGGCAGCA-3′)/806R (5′-GGACTACNNGGGTATCTAAT-3′) for the bacterial community at the V3–V4 region. The amplification was performed by the method proposed by Wei et al. (2018). High throughput sequencing was performed on Illumina Hiseq 2500 sequencing platform (Illumina, CA, United States) of Beijing Baimaike Biotechnology Co., Ltd., Beijing, China. Low quality reads were removed by Trimmonatic software (version 0.33), and FLASH (version 1.2.11) was used for combining filtered reads. The operational taxonomic units (OTUs) were obtained by integrating the sequence (Yu et al., 2018). The raw sequence was stored in the NCBI SRA database under the accession number SUB8880721.

### Statistical Analysis

Histogram, pie chart and line chart were created using Origin 9.0 (OriginLab, United States). Bubble chart was employed to explore the composition of microbial communities using R package ggplot 2. The interaction between microbial communities and molecular substantial composition was analyzed using Gephi and R 4.1.1.

## Results

### Variations in Physico–Chemical Parameters in the EFS

**FIGURE 1:**
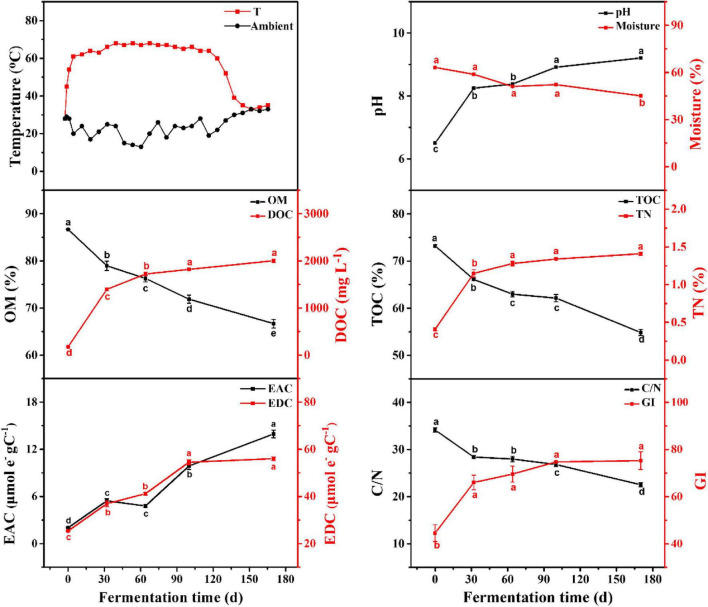
Physico-chemical parameters of samples during ectopic fermentation systems (EFS). Line plots that do not share a letter are significantly different (*p* < 0.05).

The dynamic changes in physico–chemical parameters are shown in Figure 1. According to the temperature variation, the EFS was divided into heating period (HP; days 0–32) and continuous thermophilic period (CTP; days 32–170). The moisture continued to decline, reflecting the intense mineralization in EFS. As the EFS proceeded, the contents in OM and TOC continuously reduced from 86.67 to 66.68% and from 73.21 to 54.87%, respectively. Meanwhile, the DOC concentration increased from 176.96 mg L^–1^ (day 0) to 2003.05 mg L^–1^ (day 170). During the EFS process, a continuously increasing trend existed in EDC and EAC, and the value of EDC was 4–12 times higher than that of EAC. The C/N rapidly declined from 103.70 to 21.32. Meanwhile, the GI increased gradually during EFS, and reached the maximum (75.30%) on day 170.

### Transformation of Substances in the EFS

#### Variations in Molecular Weight in EFS

**FIGURE 2:**
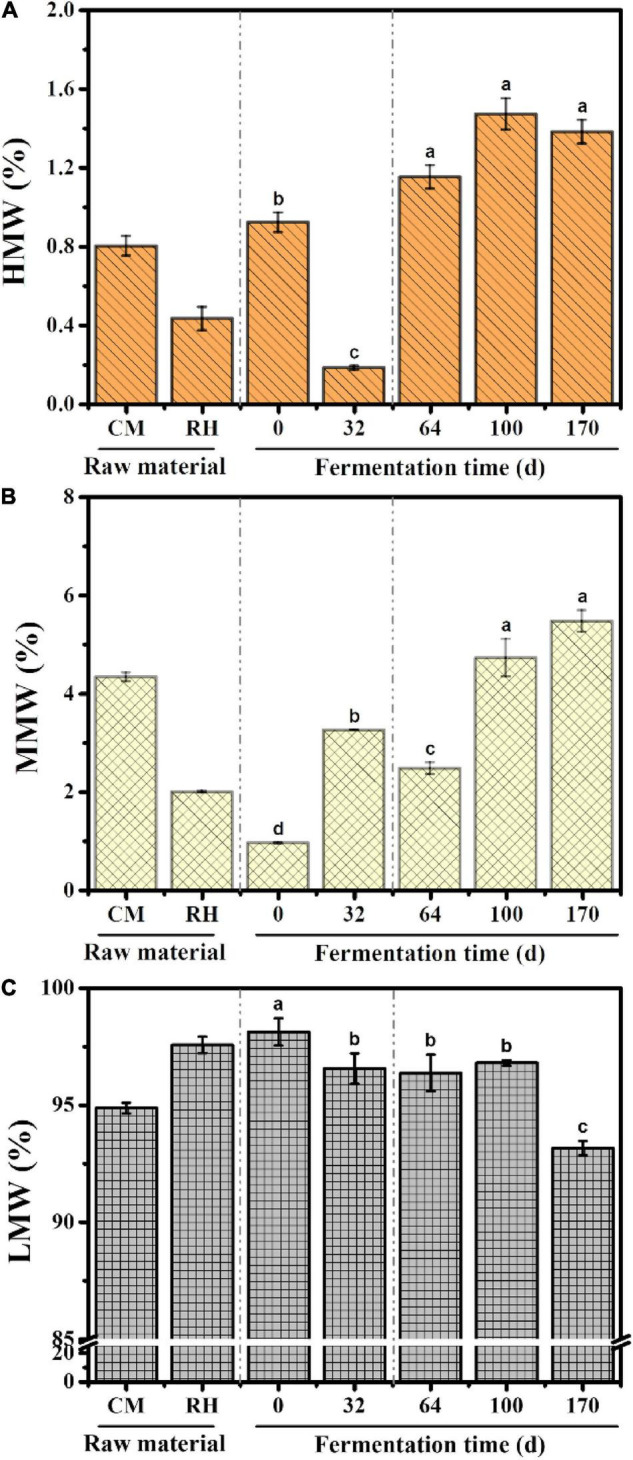
Dynamic changes in the content of different molecular weight fractions during ectopic fermentation systems (EFS): **(A)** Higher molecular weight (HMW); **(B)** Medium molecular weight (MMW); **(C)** Lower molecular weight (LMW). Histograms that do not share a letter are significantly different (*p* < 0.05).

The distribution properties of the molecular weight were obtained using peak–fitting technique. The dynamic changes in the molecular weight relative areas are shown in Figure 2. LMW fractions were the predominant fractions during EFS, followed by MMW fractions. The content of LMW fractions decreased gradually from 98.14 to 93.16% throughout the treatment. Different from LMW fractions, the content of MMW and HMW fractions showed an increasing trend, and reached the maximum 5.47 and 1.38% on day 170, respectively.

#### Variations in Composition Characteristics in the EFS

**FIGURE 3:**
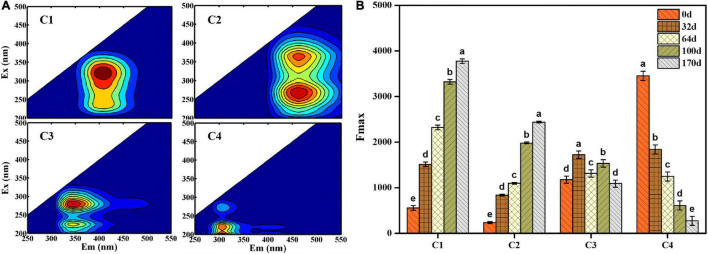
**(A)** Four fluorescence components obtained by excitation-emission matrix fluorescence combined with parallel factor analysis. **(B)** The maximum fluorescence intensity (Fmax) of four fluorescence components identified by parallel factor analysis. Histograms that do not share a letter are significantly different (*p* < 0.05).

EEM fluorescence spectra were divided into four fluorescence components detected using EEM-PARAFAC analysis: component 1 (C1) (Ex/Em = 325/410 nm), component 2 (C2) [Ex/Em = (275, 375)/420 nm, component 3 (C3) [Ex/Em = (225, 275)/340 nm] and component 4 (C4) [Ex/Em = (225, 275)/310 nm] (Figure 3A). According to the composition derived from different ecosystems (He et al., 2019; Yu Z. et al., 2019), C1 and C2 were primarily ascribed to humus-like substances, and C3 (Ex/Em = 225/340 nm) and C4 (Ex/Em = 225/310 nm) were associated with protein-like substances. More specifically, C3 was characterized by tryptophan-like substances, whereas C4 was attributed to tyrosine-like substances. In addition, C3 (Ex/Em = 275/340 nm) and C4 (Ex/Em = 275/310 nm) were identified as microbial by-products. As shown in Figure 3B, the maximum fluorescence intensity (*F*_max_) was used to analyze the distribution of four components quantitatively. The *F*_max_ values of C1 and C2 shared an increasing trend and reached a maximum value on day 170 (*F*_max_ = 3775.16 and *F*_max_ = 2400.19, respectively), indicating that EFS was a constant humification process. The *F*_max_ of C3 and C4 decreased from 1176.71 to 660.70 and 3454.54 to 233.52 during EFS, respectively.

#### Variations in Structural Properties in the EFS

**FIGURE 4:**
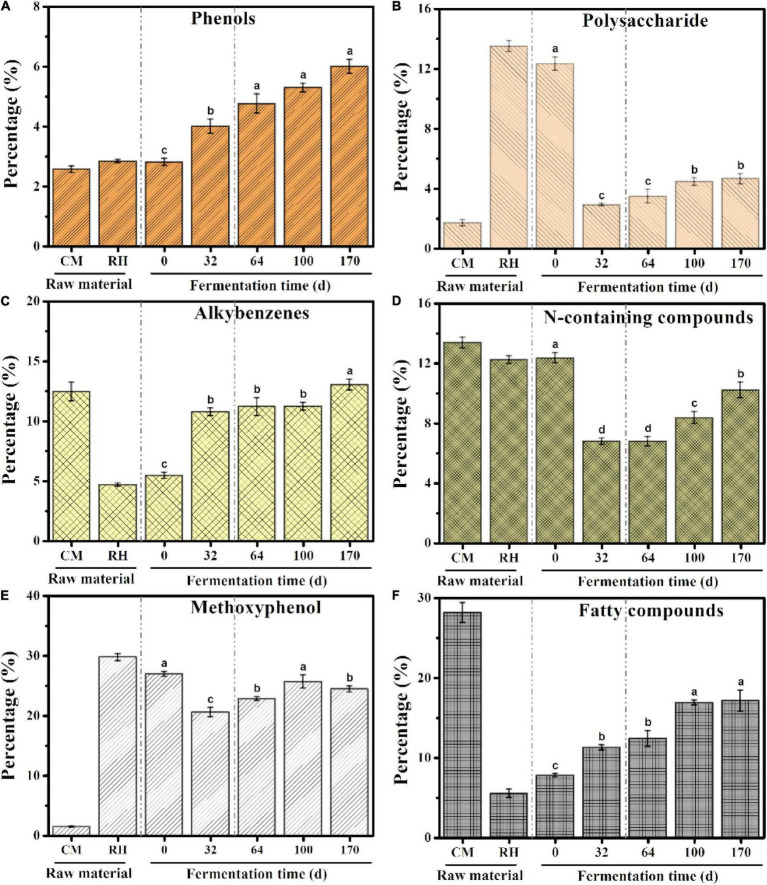
Cumulative abundance for the main groups of pyrolysates during ectopic fermentation systems (EFS): **(A)** Phenols; **(B)** Polysaccharide; **(C)** Alkybenzenes; **(D)** N-containing compounds; **(E)** Methoxyphenol; **(F)** Fatty compounds. Histograms that do not share a letter are significantly different (*p* < 0.05).

A total of 158 pyrolysates were obtained, based on the GC retention time and compared mass spectra with published and stored data (Table 1). According to the similarity in the chemical structure, the quantitative pyrolysis products were classified into six groups: phenols, polysaccharides, alkylbenzenes, N-containing compounds, methoxyphenol, and fatty compounds (Dorado et al., 2016; Zhang et al., 2019; Figure 4). Methoxyphenol was the predominant pyrolysis product in rice husk, followed by polysaccharides, which reached 29.76 and 13.51%, respectively. These resulted in higher contents of methoxyphenol and polysaccharides in the initial sample of EFS, which reached 26.92 and 12.33%, respectively. The dominant group in chicken manure was fatty compounds accounting for 28.16%, including C_13_–C_22_ lipids and their derivatives. Alkylbenzene accounted for a high proportion of the chicken manure. Toluene and styrene, which belonged to alkylbenzenes, were considered to the pyrolysis products of lignin, although styrene had multiple resources, including peptides, non-hydrolytic proteins and tannins (Dignac et al., 2006). The content of N-containing compounds in the chicken manure and rice husk was high, including indole, amine, pyrrole and pyridine, which accounted for 13.38 and 12.24%, respectively. In HP, the abundant carbon source and the insufficient nitrogen source led to the imbalance of C/N in the system. The dominant groups such as methoxyphenol and polysaccharides rapidly degraded from 26.92 to 20.52%, and 12.34 to 2.90%, respectively. Meanwhile, the content of N-containing compounds also rapidly decreased from 12.35 to 6.79%. In CTP, the C/N reached dynamic equilibrium due to the constant addition of chicken manure. Compared with HP, the contents of methoxyphenol, polysaccharides, and N-containing compounds continued to increase, with the range from 20.52 to 24.41%, 2.90 to 4.64% and 6.79 to 10.21%, respectively; and the final content (day 170) was lower than the initial content (day 0). In EFS, the content of phenols rose continuously from 2.80 to 6.00%.

**TABLE 1:** List of pyrolysates released from samples in ectopic fermentation systems (EFS).

Compounds	Peak ID	RT(min)	m/z
	**Methoxyphenol**		
Phenol, 2-methoxy-	Lg1	10.23, 11.16	109
2-methoxy-6-methylphenol	Lg2	12.12	123
Creosol	Lg3	12.41, 12.44	123
Phenol, 4-ethyl-2-methoxy-	Lg4	14.17, 19.16, 12.12	123, 137
2-methoxy-4-vinylphenol	Lg5	14.88, 15.16	135, 150
3-allyl-6-methoxyphenol	Lg6	15.75	135
Phenol, 2-methoxy-4-propyl-	Lg7	15.89, 15.92	137
Phenol, 2-methoxy-4-(1-propenyl)-	Lg8	17.47, 16.68	164
Eugenol	Lg9	15.72, 17.76	164
p-Cresol	Lg10	9.56, 10.03	107
Phenol, 2-methoxy-3-methyl-	Lg11	12.12	123
4-(1-Hydroxyallyl)-2-methoxyphenol	Lg12	19.19	137
Phenol, 2,6-dimethoxy-4-(2-propenyl)-	Lg13	21.75, 20.10	194
Phenol, 4-(ethoxymethyl)-2-methoxy	Lg13	20.92	137
(E)-2,6-dimethoxy-4-(prop-1-en-1-yl)phenol	Lg14	20.92, 21.71	194
Phenol, 2,6-dimethoxy-	Lg15	15.62	154
Phenol, 4-methoxy-3-(methoxymethyl)	Lg16	17.38	168
phenol, 2-(bromomethyl)-5-methoxy-	Lg17	32.14	137
Phenol, 4-[[2-(3,4-dimethoxyphenyl)ethylamino]methyl]-2-methoxy-	Lg18	31.41	137
	**Phenols**		
Phenol	Ph1	7.94, 8.16	94
Phenol, 2-methyl-	Ph2	9.50, 9.51, 9.62	108
Phenol, 3-methyl-	Ph3	10.03, 12.21	108
4-tert-Amylphenol	Ph4	34.30	207.10
Phenol, 3-ethyl-	Ph5	12.01	107
4-tert-butylphenol	Ph6	34.73	207
Phenol, 2-(2-methylpropyl)-	Ph7	25.07	107
3,4-Diethylphenol	Ph8	14.90	43
Phenol, 4-ethyl-	Ph9	11.91, 12.03	107
Phenol, 2,4-dimethyl-	Ph10	11.51	107
	**Alkylbenzenes**		
Benzene	B1	2.56, 2.65, 2.70, 2.42, 2.48	78
Toluene	B2	3.74, 3.77, 3.70, 3.58	91
1H-indene, 1-methylene-	B3	7.41, 12.27	106
Naphthalene, 1-methyl-	B4	14.54	142
Biphenyl	B5	16.16, 16.17	154
1,1′-biphenyl, 2-methyl-	B6	16.51	168
Naphthalene, 1,6-dimethyl-	B7	17.00	158
2,2′-Dimethylbiphenyl	B8	18.94	167.10
Benzene, nonyl-	B9	19.48	92
Naphthalene, 1,4,5-trimethyl-	B10	19.92	155.10
Benzene, undecyl-	B11	22.82	92
Phenanthrene	B12	23.17	178.10
1H-indene, 2,3,4,7-tetrahydro-	B13	13.73, 13.51, 13.73	91
Ethylbenzene	B14	5.33	91
p-Xylene	B15	5.49, 5.49, 5.36	91
3-(2-Methyl-propenyl)-1H-indene	B16	19.94	155
Benzene, 1,3-dimethyl-	B17	5.33	91
Benzene, 1-ethynyl-4-methyl-	B18	9.24	115
fluorene	B19	16.51	81
Benzene, 1-ethenyl-3-methyl-	B20	20.69	55
o-Xylene	B21	5.49	91
Styrene	B22	5.92, 5.81	104
Anthracene	B23	34.15	207
Benzene, *n*-butyl-	B24	9.44	91
Benzene, (1-ethyl-1-propenyl)-	B25	13.02	117
Benzene, pentyl-	B26	11.62	91
Benzene, hexyl-	B27	13.73	91
Benzene, heptyl-	B28	15.75	91
Benzene, octyl-	B29	17.66	92
4-Ethylbiphenyl	B30	18.79	167
	**N-containing compounds**		
4-Cyanocyclohexene	N1	4.28	54
Indol-2-one, 3-amino-1,3-dihydro-	N2	14.01	120
1H-Pyrrole-2,5-dicarbonitrile	N3	14.82, 17.10, 19.27, 20.70, 8.83	117, 137, 91
4-Nitrocatechol	N4	12.21	82
Pyridine, 5-ethenyl-2-methyl-	N5	21.10	91
1H-Indole, 1-methyl-2-phenyl-	N6	34.60, 34.15	207
1,2-Benzisothiazol-3-amine	N7	35.28	207
Pyrazole	N8	4.81	95
Indole	N9	14.65, 14.75, 14.81	117
Pyrimidine, 5-methyl-	N10	8.38	94
Pyrazine, 2-methoxy-3-(1-methyleth yl)-	N11	14.30	137
3-Dimethylaminoacrylonitrile	N12	5.150	96
biphenylene	N13	36.07	207
Benzo[b]thiophene	N14	18.00, 18.12, 37.18, 37.90,	147
1H-Imidazole, 4,5-dimethyl-	N15	4.48	96
Pyrazole	N16	4.84	95
Benzo[b]thiophene	N17	18.00, 18.12	147
Butanenitrile	N18	2.18	54
1H-Pyrrole, 1-methyl-	N19	3.42, 3.43	81
1H-Pyrrole, 3-methyl-	N20	5.12	80
Benzyl nitrile	N21	11.38	117
Benzenepropanenitrile	N22	13.50	91
Indolizine	N23	14.60, 14.65	117
1H-Indole, 2-methyl-	N24	16.46	130
Hexadecanenitrile	N25	22.52, 24.61	43
Pentadecanenitrile	N26	23.10	43
2-Methyl-7-phenylindole	N27	37.95	207
5-Methyl-2-phenylindolizine	N28	38.07	207
Pyrrolo[1,2-a]pyrazine	N29	8.14	118
Benzo[h]quinoline	N30	35.68, 36.43, 36.94	207
5-Methyl-2-phenylindolizine	N31	35.86	207
1,4-Phthalazinedione, 2,3-dihydro-6-nitro-	N32	38.06	207
Pyridine	N33	3.58	81
1H-Pyrazole, 3,4-dimethyl-	N34	4.94	79
1H-Pyrrole, 3-ethyl-	N35	5.13	80
Benzenamine, 3-methoxy-	N36	12.60	123
Benzenamine, N, 4-dimethyl-	N37	14.06	91
	**Polysaccharide**		
Benzofuran, 2,3-dihydro-	Ps1	13.16, 13.57	120
Furan, 2-(3-imino-3-ethoxyprop-1-enyl)-	Ps2	18.57	91
1,4-dimethyl-2,4-dimethylfuran	Ps3	5.00	96
Furan, 2-methyl-	Ps4	2.27, 2.26	82
Furan, 2,5-dimethyl-	Ps5	5.26, 3.02	96
beta.-D-Mannofuranoside, farnesyl	Ps6	22.17	69
Benzofuran	Ps7	13.19	120
3-Methyl-2-(2-methyl-2-butenyl)-furan	Ps8	15.26	150.10
7-Benzofuranol, 2,3-dihydro-2,2-dimethyl-	Ps9	17.76	105.10
2,4-Dimethylfuran	Ps10	4.95	82
Benzofuran, 2,3-dihydro-	Ps11	12.99	164
D-Allose	Ps12	18.57	60
beta.-D-Glucopyranose, 1, 6-anhydro-	Ps13	18.70	60
2-Vinylfuran	Ps14	3.19	94
Carbofuran	Ps15	16.56	164
isobenzofuran	Ps16	34.34	298
	**Fatty compounds**		
1-Tridecene	Fc1	14.30	43
Tridecane	Fc2	14.46, 19.88	57
Tetradecene	Fc3	16.22	55
Tetradecane	Fc4	16.36	57
Pentadecane	Fc5	18.17	57
Heptadecene	Fc6	21.40	55
Heptadecene	Fc7	21.51	57
Hexadecene	Fc8	21.99	65
Hexadecanoic acid, 2-methyl-	Fc9	24.39	74
3-Methylhexacosane	Fc10	24.52	57
Hexadecanoic acid, methyl ester	Fc11	24.93	74
*n*-Hexadecanoic acid	Fc12	25.56	55.1
Docosane	Fc13	25.92	57.1
11-Octadecenoic acid, methyl ester	Fc14	27.47	55
Heptadecanoic acid, 16-methyl-thyl ester	Fc15	27.66	74
1-Pentadecene	Fc16	18.04	43
Hexatriacontane	Fc17	19.88	57
Z,Z-2,15-Octadecedien-1-ol acetate	Fc18	6.79	41
Spiro[2.5]octane-1,1-dicarbonitril	Fc19	25.44	55
Cyclododecane	Fc20	12.27	55
1-Tetradecene	Fc21	16.22	55
Octadecene	Fc22	22.95	55
1-Hexadecanol, 2-methyl-	Fc23	24.06	55
Non-adecane	Fc24	24.52	57.10
Decanynoic acid	Fc25	25.24	55
Hexadecane	Fc26	25.93	57
Octadecane	Fc27	27.26	57
Heptadecanoic acid	Fc28	27.65	91
Eicosane	Fc29	28.56	57
1, 9-Tetradecadiene	Fc30	28.14	55
Limonene	Fc31	8.83	68
Androstane	Fc32	9.68	41
Cyclododecane	Fc33	13.02	67
Endo-tricyclo[5.2.1.0(2.6)]decane	Fc34	12.60	95
Hexadecane	Fc35	19.88	57
9-Tricosene, (Z)-	Fc36	22.95	55
Non-adecane	Fc37	24.52	57
9-Undecen-2-one, 6,10-dimethyl	Fc38	25.52	43
Z-5-Non-adecene	Fc39	27.19	55
1,9-Tetradecadiene	Fc40	28.14	55
Heneicosane	Fc41	27.27	57
Cyclotetradecane	Fc42	14.30	53
Cyclododecanediol	Fc43	15.93	55
Dodecane	Fc44	12.44	57
Eicosane	Fc45	25.93	57
Cholest-2-ene	Fc46	35.48	43
Cyclopentadecanone, 2-hydroxy	Fc47	27.83	55

### Succession of the Bacterial Community in the EFS

**FIGURE 5:**
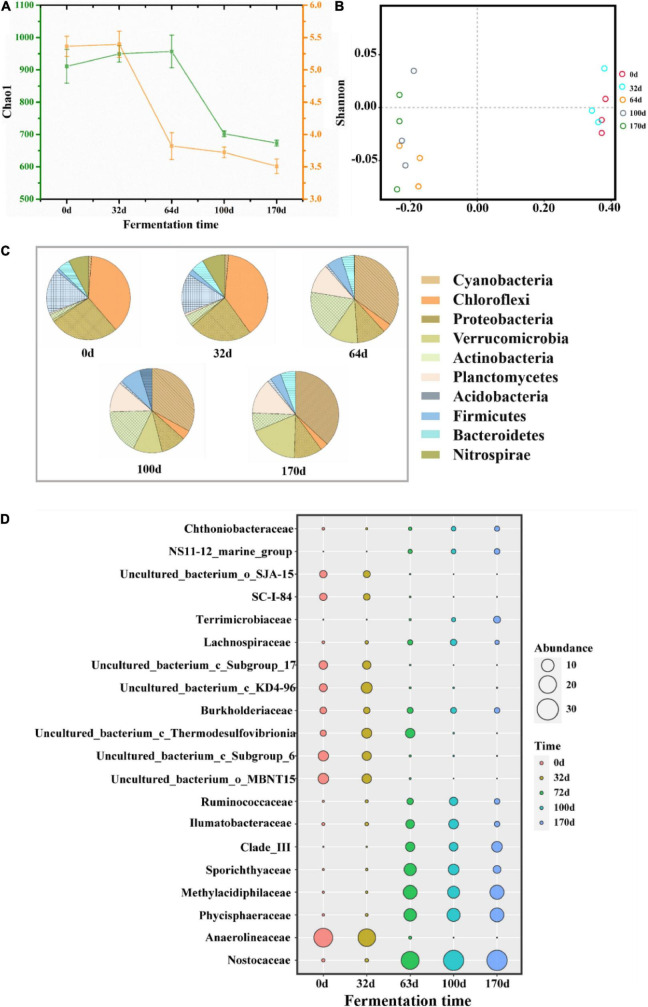
Taxonomic classification at different levels of predominant 16S rRNA sequencing in samples during ectopic fermentation systems (EFS). **(A)** Alpha diversity; **(B)** PCoA analysis; **(C)** Phylum level (relative abundance at top 10); **(D)** Family level (relative abundance at top 20).

The changes in bacterial community diversity in different stages were compared by analyzing alpha and beta diversity. As shown in Figure 5A, both Chao1 and Shannon indices showed a decreasing trend, especially in CTP. This finding likely stemmed from the decrease in bacterial abundance and diversity caused by the continuous thermophilic environment during CTP. In addition, based on PCoA analysis, the bacterial structure (at the OTU level) was found to change intensively between HP and CTP (Figure 5B). The taxonomic classification at the phylum (relative abundance at top 10) and family (relative abundance at top 20) levels are presented in Figures 5C,D, respectively. The dominant bacterial phyla were *Cyanobacteria*, *Chloroflexi*, *Proteobacteria*, *Verrucomicrobia*, *Actinobacteria*, *Planctomycetes*, *Acidobacteria* and *Firmicutes*, which accounted for 78.05–94.26% during EFS and were usually detected in composting, soil and freshwater (Yamada et al., 2008; Li et al., 2019; Yu H. M. et al., 2019). The dynamic changes in microbial communities exhibited apparent discrepancy as the EFS proceeded. In HP, the predominant bacterial communities were distinguished as *Chloroflexi*, followed by *Proteobacteria* and *Acidobacteria*. However, the dominant bacterial communities shifted to *Cyanobacteria*, *Actinobacteria* and *Planctomycetes* in CTP. These results showed that the changes in temperature had a significant effect on microbial composition. The abundance of *Nostocaceae* and *Cyanobacteria* increased significantly in CTP, and relative abundance ranges were 0.54–35.00 and 1.09–37.30%, respectively. *Planctomycetes* could adapt to the thermophilic environment, and abundancemainly increased (0.55–12.85%) in CTP (Kovaleva et al., 2015). The family *Phycisphaeraceae*, belonging to *Planctomycetes*, increased from 0.25 to 13.83%. The abundance of *Actinobacteria* ascended rapidly in CTP with a maximum of 17.09%. The relative abundance of *Firmicutes* increased to 7.99% in CTP. The relative abundance of *Chloroflexi* significantly decreased from 33.45 to 8.03%, and a similar tendency was detected for *Anaerolineaceae* decreasing from 23.78 to 0.01%.

### Correlations Between Microbial Communities and Substance Transformation in the EFS

#### Correlations Between Substance Transformation and Maturity

**FIGURE 6:**
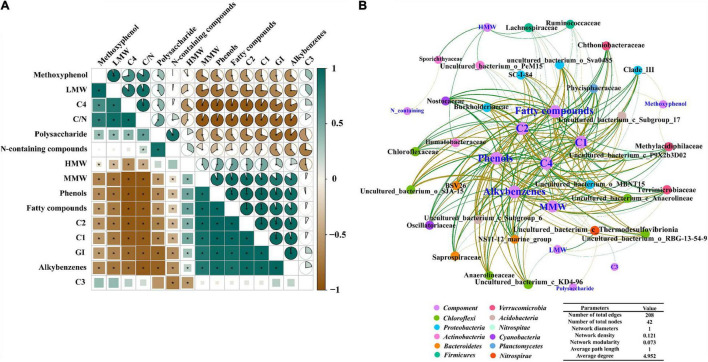
**(A)** The correlation between substances transformation and maturity of ectopic fermentation systems (EFS; *ρ-value > 0.8, *P* < 0.05); **(B)** Molecular ecological network analysis of cooccurring bacterial community and molecular composition in EFS.

As shown in Figure 6A, HMW, MMW, C1, and C2 significantly correlated with each other; all of them showed a significant negative correlation with LMW and C4. Phenols, fatty compounds and alkylbenzenes were significantly positively related to C1 (*P* < 0.01) and C2 (*P* < 0.01), while these compositions showed a negative correlation with C4 (*P* < 0.001). GI was significantly positively correlated with humus-like substance such as C1, C2, and MMW. In addition, it was apparent that polysaccharide, C4 and LMW negatively correlated with GI. Meanwhile, GI shared positive correlations with phenols (*P* < 0.001), while a significant negative correlation was found between phenols and C/N. Contrary to GI, C/N exhibited a positive correlation with LWM and C4.

#### Characteristics of the Molecular Network Between Substance Transformation and Bacterial Communities

As can be seen from Figure 6B, the number of network nodes and edges was 42 and 208, respectively. In the molecular network, 42 nodes were classified into 12 groups based on the phylum. Groups *Chloroflexi*, *Proteobacteria*, *Actinobacteria*, *Bacteroidetes* and *Verrucomicrobia* accounted for 47.61% of the total nodes, demonstrating that the changes in these bacterial communities affected the substance transformation to a greater extent. Generally consistent positive and negative associations (103 vs 105) were observed in the study, suggesting that the potential competition and mutualistic correlation between microbial communities and different molecular compositions were mutually balanced. HMW had positive associations with some taxa (e.g., *Sporichthyaceae*, *Ilumatobacteraceae*, *Ruminococcaceae*, and *Lachnospiraceae*). Meanwhile, *Nostocaceae* (*Cyanobacteria*), *Phycisphaeraceae* (*Planctomycetes*), *Chloroflexaceae* (*Chloroflexi*) and *Oscillatoriaceae* (*Cyanobacteria*) were positively associated with phenols as detected by the network analysis. Besides, phenols, these thermotolerant bacterial communities were associated with C1 and C2 and showed a negative correlation with C4.

## Discussion

The EFS kept the thermophilic temperature (>50°C) for 160 days, which might be due to the thermophilic inoculants to promot the degradation of organic substances. In a hyperthermal environment, the content of organic matter decreased. It was speculated that drastic organic substances degradation took place in EFS, where OM and TOC were converted to DOC (Yu et al., 2018). The enhancement of EAC may be caused by the continuous formation of oxidation functional groups in aromatic compounds during EFS. Meanwhile, the increase in EDC might be related to the reducing groups produced by the transformation of organic matter. Previous studies demonstrated that the C/N and GI were mainly used as indices to evaluate the compost maturity (Zeng et al., 2009; Yu et al., 2011). The C/N decreased gradually, demonstrating that the carbon resource from the husk was continuously consumed during EFS. The GI value ascended gradually with the composting, and reached the maximum (75.30%) on day 170. Chen et al. (2005) proposed that a GI value higher than 50% implied toxicity-free. These results demonstrated that EFS was the continuous maturation process and the final sample was no–toxic.

The dynamic changes in molecular weight and composition of substances exhibited apparent discrepancy as the EFS proceeded. The decline in LMW might be due to the high microbial activity, that is, small–molecule–substances were constantly decomposed and polymerized by microorganisms. Nevertheless, contradictory trends were seen in MMW and HMW. On the one hand, microorganisms continue to decompose easily degradable organic matter, leaving refractory substances that are inert to microorganisms. On the other hand, functional microorganisms are devoted to synthesizing macromolecular organic matter from small molecular substances in rice husk, chicken manure, and by-products of microbial decomposition. These results demonstrated that the aromatization and stability of samples were enhanced. HMW fractions had strong stability, and the increase of their content was beneficial to improve soil properties and accelerate the transformation of pollutants (Steinberg and Regan, 2008; Huang et al., 2021). The contents of HMW and MMW fractions in CTP were much higher than those in HP. The EEM fluorescence spectra revealed that the substance transformation in EFS was obvious. The increased *F*_max_ of C1 and C2 and decreased *F*_max_ of C3 and C4 further indicated that the thermophilic environment could accelerate the degradation of protein-like substances (from 4631.25 to 894.22) and the formation of humus–like substances (from 793.78 to 6215.35) in EFS. Combined with molecular weight and fluorescence components, an evident transformation was observed from small–molecule substances such as protein-like substances to the large–molecule substances such as humus-like substances during EFS. The phenols, which could indicate maturity, have a great potential in complexing heavy metals and enhance electron transfer capacity in composting (Tang et al., 2019; Huang et al., 2021). A continuously increasing trend for phenols indicated that the samples gradually tended to mature in a thermophilic environment. Under the dynamic balance of carbon and nitrogen and continuously high temperature, the polycondensation of humus precursors and small–molecule compounds were more obvious, compared with the degradation of substances. Based on the aforementioned conclusions, it was presumed that the addition of chicken manure promoted the continuous thermophilic environment and the transformation of substances in EFS, which was mainly devoted to regulating the carbon–nitrogen balance to degrade substances and accelerate the synthesis of humus precursor phenols.

The bacterial communities shifted significantly as EFS proceeded. As the most predominant microbial community, *Cyanobacteria* was found in freshwater lakes (Yilmaz et al., 2008), but rarely reported in EFS. The family *Nostocaceae* within the phylum *Cyanobacteria* could survive in a wide range of temperature and nutrient concentrations, and high temperature could promote their growth (Paerl et al., 2001; Antunes et al., 2015). Earlier investigations demonstrated that *Phycisphaeraceae* was involved in the global nitrogen cycle (Rios-Del Toro et al., 2018) and could degrade complex refractory substances (Liang et al., 2019). These bacteria might maintain a thermophilic environment and degrade refractory substances in EFS. *Actinobacteria* are thermotolerant; they can survive at high temperatures and produce antibiotics for killing pathogenic microorganisms (Li et al., 2019). *Firmicutes* are important bacteria with high utilization of carbohydrates; they also resist high temperature (Haakensen et al., 2008; Tian and Gao, 2014). *Ruminococcaceae* have been reported to survive in fibrinolytic communities and contribute to the degradation of refractory substances. *Chloroflexi* are detected in various ecosystems such as soil and sludge; most of them exist in mesophilic and thermophilic environment (Yamada et al., 2006; Kim et al., 2019). *Anaerolineaceae* (within phylum *Chloroflexi*) could promote the degradation of organic substances by taking advantage of carbohydrates and proteinaceous carbon sources (Bovio et al., 2019). In summary, distinct discrepancies exist in the distribution of dominant bacterial communities during EFS. In HP, the dominant bacterial communities might be inclined to break down organic substances. In CTP, thermotolerant bacterial communities such as *Cyanobacteria* (*Nostocaceae*), *Planctomycetes* (*Phycisphaeraceae*), *Actinobacteria* and *Firmicutes* played a vital role in sustaining the thermophilic environment, promoting the transformation of refractory substances and accelerating the maturation of EFS.

The correlation analysis of molecular weight, composition characteristics, structural properties and maturity was conducted to clearly understand the substance transformation and its effect on the maturation during EFS. Humus–like substances significantly negatively correlated with protein–like substances, indicating that the formation of humus-like substances was caused by the condensation of large–molecule fractions and the degradation of small–molecule fractions. Phenols, fatty compounds and alkylbenzenes positively correlated with C1 but negatively correlated with C4, revealing that phenols, fatty compounds and alkylbenzenes might play an important role in the humification during EFS. As an index to evaluate maturity, GI had correlation with phenols. Yuan et al. (2012) found that phenols could be used as a marker to indicate maturity, which was in agreement with our results. These results showed that substance transformation was closely related to the maturity process. The continuous thermophilic environment accelerated the material transformation process, such as the rapid formation of phenol, resulting in the fast maturation in EFS.

The molecular network analysis indicated that *Sporichthyaceae*, *Ilumatobacteraceae*, *Ruminococcaceae* and *Lachnospiraceae* belonging to *Actinobacteria* and *Firmicutes* positively correlated with HMW, suggesting that *Actinobacteria* and *Firmicutes* degrading cellulose might promote the generation of HMW. In addition, thermotolerant bacterial communities, *such as Nostocaceae and Phycisphaeraceae*, were associated with phenols, suggesting that these thermotolerant bacterial communities might contribute to the formation of phenols in EFS. The thermotolerant bacterial communities had a positive correlation with humus–like substances, and a negative correlation with protein–like substances. These results suggested that the thermotolerant bacterial communities might promote the transformation of protein–like small–molecule compounds to humus–like large–molecule compounds. In conclusion, thermotolerant bacterial communities were crucial to phenol formation and could accelerate humification by degrading protein–like substances and synthesizing humus-like substances.

EFS was in the thermophilic phase for a long time. In addition, EFS could deal with massive solid waste, processing about 850 t of chicken manure. These show that EFS is an efficient and economical way to treat solid waste. Thermotolerant bacteria were functional bacteria that promoted the degradation of protein-like substances and the generation of humus-like substances, thus promoting humification. The aromatic substances phenols continuously generated in this process can better indicate maturity.

## Conclusion

The EFS lasted for 170 days and sustained the thermophilic temperature (>50°C) for 160 days. The HMW fractions (from 0.18 to 1.47%), humus-like substances (from 793.78 to 6215.35), and phenols (from 2.80 to 6.00%) were rapidly formed during EFS. Thermotolerant bacterial communities (from 0.79 to 48.83%), such as *Nostocaceae* and *Phycisphaeraceae*, were dominant in CTP and contributed to thermophilic environment, acceleration of humification, and formation of phenols. In addition, phenols could be used as an indicator of the maturity of EFS. This study helped to advance our understanding of the biochemical transformation during EFS.

## Data Availability Statement

The raw sequence was stored in the NCBI SRA database under the accession number SUB8880721.

## Author Contributions

PW, TC, and ZY: conceptualization. PW and WH: methodology. PW, WH, and WW: investigation. PW: writing – original draft. PW, WH, YW, TC, and ZY: writing – review and editing. YW and ZY: funding acquisition. All authors read and approved the manuscript.

## Conflict of Interest

The authors declare that the research was conducted in the absence of any commercial or financial relationships that could be construed as a potential conflict of interest.

## Publisher’s Note

All claims expressed in this article are solely those of the authors and do not necessarily represent those of their affiliated organizations, or those of the publisher, the editors and the reviewers. Any product that may be evaluated in this article, or claim that may be made by its manufacturer, is not guaranteed or endorsed by the publisher.

## Footnotes

1
http://www.models.kvl.dk/
