# Supplementary material for: Linking Microbial Community Succession With Substance Transformation in a Thermophilic Ectopic Fermentation System
Source: Front Microbiol. 2022 May 4;13:886161. doi: 10.3389/fmicb.2022.886161 (PMC9116721; doi:10.3389/fmicb.2022.886161)
Supplement: Supplementary file 1 [file Table_1.DOCX]

Table captions

**Table S1.** Comparison of predominant phylogenetic groups in different fermentation systems.

**Table S2.** The predominant family in ectopic fermentation systems (EFS).

Table S1 Comparison of predominant phylogenetic groups in different fermentation systems

|  | **Materials** | **Predominant phylogenetic groups** | | | ***References*** |
| --- | --- | --- | --- | --- | --- |
|  |  | **Phylum level** | **Family level** | **Genus level** |  |
| 1 | rice husk and  chicken manure | *Cyanobacteria,*  *Planctomycetes* | *Nostocaceae,*  *Phycisphaeraceae,* | *-* | *this study* |
| 2 | agricultural byproducts and  pig manure | *Firmicutes, Actinobacteria,*  *Proteobacteria,*  *Bacteroidetes* | *-* | *Corynebacterium,*  *Bacillus,*  *Virgibacillus,*  *Pseudomonas,*  *Actinobacteria,*  *Lactobacillus* | *Chen et al., 2017* |
| 3 | cow dung | *Firmicutes* | *Enterobacteriaceae* | *Bacillus* | *Yang et al., 2018* |
| 4 | mattress materials and pig manure | *Proteobacteria,*  *Actinobacteria,*  *Bacteroidetes* | *-* | *Acinetobacter,*  *Pseudomonas,* | *Chen et al., 2020* |
| 5 | Maize straw,  Mushroom,  Sawdust and cow wastewater | *Firmicutes, Verrucomicrobia,*  *Proteobacteria, Actinomycetes* | *-* | *Bacillus* | *Guo et al., 2015* |
| 6 | pig manure | *Proteobacteria,*  *Bacteroidetes,*  *Firmicutes* | *-* | *Chitinophaga, Bacteroides,*  *Serratia, Stenotrophomonas,*  *Pseudomonas,*  *Anoxybacillus* | *Shen et al., 2019* |

-, not available.

*References:*

[1] Chen Q, Liu B, Wang J, et al. Diversity and dynamics of the bacterial community involved in pig manure biodegradation in a microbial fermentation bed system [J]. *Ann Microbiol*, 2017, 67(7): 491-500.

[2] Yang X, Geng B, Zhu C, et al. Fermentation performance optimization in an ectopic fermentation system [J]. *Bioresour technol,* 2018, 260: 329-337.

[3] Chen Q, Wang J, Zhang H, et al. Microbial community and function in nitrogen transformation of ectopic fermentation bed system for pig manure composting [J]. *Bioresour Technol*, 2021, 319: 124155.

[4] Guo H, Zhu C, Geng B, et al. Improved fermentation performance in an expanded ectopic fermentation system inoculated with thermophilic bacteria [J]. *Bioresour Technol*, 2015, 198: 867-875.

[5] Shen Q, Sun H, Yao X, et al. A comparative study of pig manure with different waste straws in an ectopic fermentation system with thermophilic bacteria during the aerobic process: Performance and microbial community dynamics [J]. *Bioresour technol*, 2019, 281: 202-208.

Table S2 The predominant family in ectopic fermentation systems (EFS)

| **Family** | **Relative abundance/ %** | **Characteristics** | **References** |
| --- | --- | --- | --- |
| *Anaerolineaceae* | 0.03-23.79% | Decomposing carbohydrates or polypeptides | Yamada et al., 2006 |
| *Methylacidiphilaceae* | 0.03-23.78% | Fixing CO_2_ | Kruse et al., 2019 |
| *Sporichthyaceae* | 0.16-9.61% | Adapting to moderately acidic environment | Lee et al., 2018 |
| *Clade_III* | 0.06-7.17% | Decomposing organic  compound | Lu et al., 2015 |
| *Ruminococcaceae* | 0.19-4.25% | Degrading cellulose and hemicellulose | Biddle et al., 2013 |

*References:*

[1] Yamada T, Sekiguchi Y, Hanada S, et al. *Anaerolinea thermolimosa* sp. nov., *Levilinea saccharolytica* gen. nov., sp. nov. and *Leptolinea tardivitalis* gen. nov., sp. nov., novel filamentous anaerobes, and description of the new classes *Anaerolineae* classis nov. and *Caldilineae* classis nov. in the bacterial phylum *Chloroflexi* [J]. Int J Syst Evol Microbiol, 2006, 56(6): 1331-1340.

[2] Kruse T, Ratnadevi C M, Erikstad H A, et al. Complete genome sequence analysis of the thermoacidophilic verrucomicrobial methanotroph “*Candidatus Methylacidiphilum kamchatkense*” strain Kam1 and comparison with its closest relatives [J]. *BMC Genom*, 2019, 20(1): 1-15.

[3] Lee D G, Trujillo M E, Kang S, et al. *Epidermidibacterium keratini* gen. nov., sp. nov., a member of the family *Sporichthyaceae*, isolated from keratin epidermis [J]. Int *J Syst Evol Microbiol*, 2018, 68(3): 745-750.

[4] Lu L, Zeng G, Fan C, et al. Environmental factors shaping the abundance and distribution of laccase-encoding bacterial community with potential phenolic oxidase capacity during composting [J]. *Appl microbiol biot*, 2015, 99(21): 9191-9201.

[5] Biddle A, Stewart L, Blanchard J, et al. Untangling the genetic basis of fibrolytic specialization by *Lachnospiraceae* and *Ruminococcaceae* in diverse gut communities [J]. *Diversity*, 2013, 5(3): 627-640.
